# A hydrogen peroxide economizer for on-demand oxygen production-assisted robust sonodynamic immunotherapy

**DOI:** 10.7150/thno.64862

**Published:** 2022-01-01

**Authors:** Qinqin Jiang, Bin Qiao, Xiaohong Lin, Jin Cao, Nan Zhang, Huanling Guo, Weiwei Liu, Lingyu Zhu, Xue Xie, Li Wan, Rui Tang, Bing Liang, Dong Wang, Zhigang Wang, Yang Zhou, HaiTao Ran, Pan Li

**Affiliations:** 1 Department of Ultrasound, Chongqing Key Laboratory of Ultrasound Molecular Imaging, the Second Affiliated Hospital of Chongqing Medical University, Chongqing 400010, P. R. China.; 2 Department of Medical Ultrasonics, The First Affiliated Hospital of Sun Yat-sen University, Guangzhou, 510080, P. R. China.; 3 Department of Ultrasound China-Japan Union Hospital of Jilin University, Jilin 130033, P. R. China.; 4 Department of Pathology, Chongqing Medical University, Chongqing, 400016, P. R. China.; 5 Department of Ultrasound, the First Affiliated Hospital of Chongqing Medical University, Chongqing 400010, China.; 6 Department of Ultrasound, the Third People's Hospital of Chengdu City, the Affiliated Hospital of Southwest Jiaotong University.

**Keywords:** sonodynamic therapy, immunotherapy, nanozyme, cancer cell membrane, focused ultrasound

## Abstract

The outcome of sonodynamic immunotherapy is significantly limited by tumor hypoxia. To overcome this obstacle, one common solution is to catalyze the conversion of endogenous H_2_O_2_ into O_2_. However, the effectiveness of this strategy is limited by the insufficient concentration of H_2_O_2_ in the tumor microenvironment (TME). Herein, we developed a H_2_O_2_ economizer for on-demand O_2_ supply and sonosensitizer-mediated reactive oxygen species production during ultrasound activation, thereby alleviating hypoxia-associated limitations and augmenting the efficacy of sonodynamic immunotherapy.

**Methods:** The H_2_O_2_ economizer is constructed by electrostatic adsorption and π-π interactions between the Fe-doped polydiaminopyridine (Fe-PDAP) nanozyme and chlorin e6. By employing a biomimetic engineering strategy with cancer cell membranes, we addressed the premature leakage issue and increased tumor-site accumulation of nanoparticles (membrane-coated Fe-PDAP/Ce6, MFC).

**Results:** The prepared MFC could significantly attenuate the catalytic activity of Fe-PDAP by reducing their contact with H_2_O_2_. Ultrasound irradiation promoted MFC dissociation and the exposure of Fe-PDAP for a more robust O_2_ supply. Moreover, the combination of MFC-enhanced sonodynamic therapy with anti-programmed cell death protein-1 antibody (aPD-1) immune checkpoint blockade induced a strong antitumor response against both primary tumors and distant tumors.

**Conclusion:** This as-prepared H_2_O_2_ economizer significantly alleviates tumor hypoxia via reducing H_2_O_2_ expenditure and that on-demand oxygen-elevated sonodynamic immunotherapy can effectively combat tumors.

## Introduction

Sonodynamic therapy (SDT) emerges as a promising site-specific tumor cells killing strategy due to the unique features with non-invasiveness, great operational feasibility, and high tissue penetration depth. It uses low-intensity ultrasound (US) as a source of stimuli to generate highly cytotoxic reactive oxygen species (ROS) *via* activation of a sonosensitizer 1-4. Recently, it has been found that SDT can release tumor-associated antigens (TAAs) and induce immunogenic cell death (ICD), which facilitate the redistribution and activation of immune effector cells with enhanced tumor-specific T cell infiltration. The combination of checkpoint blockade with SDT has shown a synergistic effect on tumor treatment.^1^ Unfortunately, such a sonodynamic immunotherapy approach is not strong enough to eliminate tumors because the highly hypoxic tumor microenvironment (TME) makes it difficult to generate robust ROS during cancer treatment, giving rise to insufficient TAAs generation and limited antitumor immune effect.^5^-^7^ Therefore, there is a pressing need to explore practical strategies for elevating the tumor oxygen content while simultaneously enhancing the benefit of sonodynamic immunotherapy.

Recently, various oxygen carriers, such as hemoglobin and perfluorocarbons, have been explored for their ability to elevate the tumor oxygen content.^8^-^10^ However, the outcomes are limited by low O_2_-loading efficiency. Alternatively, *in situ* catalysis of O_2_ generation inside tumors is a common oxygen-replenishing strategy 11-13. To this end, several O_2_-releasing nanosystems (*e.g.*, manganese dioxide nanoparticles, gold nanoclusters, and Fe^3+^-doped building blocks) have been reported 14-18; these nanosystems can be transported to tumor sites and catalyze the conversion of endogenous H_2_O_2_ into O_2_. However, these strategies suffer from insufficient concentrations of H_2_O_2_ (50-100 μM) in the TME, and their outcomes are limited.^19^, ^20^ In addition, the O_2_-evolving catalytic reaction process is usually rapid and uncontrolled. The inevitable premature consumption of H_2_O_2_ by catalase could lead to insufficient H_2_O_2_ supply during O_2_-augmented SDT, which prevents alleviation of hypoxia-related SDT resistance during the therapeutic process. Therefore, solutions that allow “broadening sources” of H_2_O_2_ in the TME are highly desirable for efficient O_2_ generation and hypoxia alleviation, which has been rarely reported.

We assume that the surface carrying agents on catalase could significantly lock the catalase-like activity due to the reduced contact with H_2_O_2_ 21, 22. Upon ultrasound irradiation, the carrying agents disintegrate from the catalase *via* the cavitation effect, and their catalytic capability is recovered 23-27. On this basis, H_2_O_2_ can be economically used through ultrasound-activatable catalase for burst-like O_2_ release when required; catalase activity activated by ultrasound can reduce premature consumption of H_2_O_2_ and induce a more adequate H_2_O_2_ supply during SDT, which in turn more efficiently potentiates O_2_ release and ROS generation. Different from previous approaches, such “lock and key” strategy could avoid many H_2_O_2_ molecules to be unavailable for O_2_ enhanced SDT because of cell respiration. Although several drugs, such as glucose oxidase 28, engineered bacteria 29, β-lapachone 30, and nanoselenium 31, have been explored for the synthesis of H_2_O_2_ *via* different mechanisms, their generation procedure requires a high level of O_2_ as a raw material, which is not well suited to SDT. For example, Dai *et al.* used a functional Dox@Pt prodrug Fe nanoparticles with nicotinamide adenine dinucleotide phosphate (NADPH) oxidases (NOXs) and superoxide dismutase (SOD) activity, which can colonize tumor regions and increase localized H_2_O_2_ generation by transferring electrons from NADPH to O_2_.^32^ In contrast to expanding the sources of H_2_O_2_, inhibiting unnecessary H_2_O_2_ expenditure may be a promising strategy to combat hypoxia before SDT treatment. Therefore, we propose a “H_2_O_2_-economizer” strategy for on-demand H_2_O_2_ decomposition-assisted O_2_ generation instead of the currently prevalent O_2_ supply approach. Such an H_2_O_2_ economizer is particularly important for enhancing SDT efficacy, which provides promising approaches to activate antitumor response 33.

**Scheme 1:**
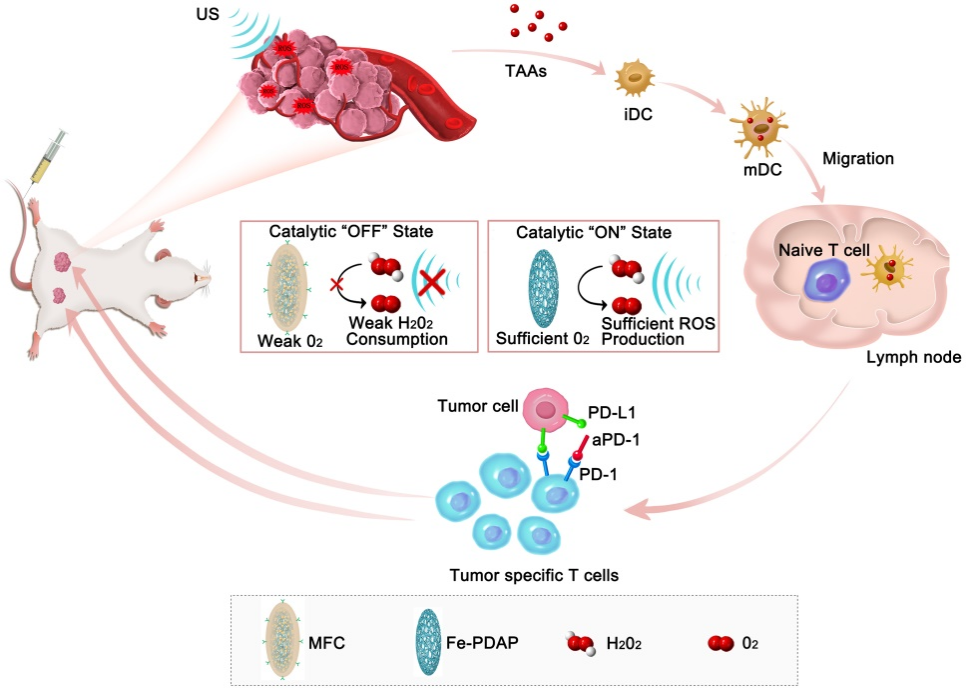
Schematic illustration of the antitumor immune responses induced by MFC-based on-demand O_2_-evolving SDT combined with immune checkpoint blockade for effective immunotherapy against cancer.

Herein, we loaded the sonosensitizer chlorin e6 (Ce6) onto catalase-like Fe-PDAP (Fe-doped polydiaminopyridine), which was subsequently coated with the cancer cell membrane to design membrane-coated Fe-PDAP/Ce6 (MFC) for sonodynamic immunotherapy. As constructed, MFC was expected to possess the following favorable properties. 1) The Fe-PDAP in the MFC could simultaneously serve as the carrier and catalase-like nanozyme. 2) MFC could perform effective sonodynamic immunotherapy, which originated from an “H_2_O_2_ economizer”-mediated on-demand O_2_ evolving process. The catalase-like activity of the MFC was significantly locked to reduce the unnecessary consumption of H_2_O_2_ in the TME. Upon irradiation with low-intensity focused ultrasound (US)^34^, ^35^, whose acoustic beam is targeted and focused, the MFC can be selectively disassembled to liberate Fe-PDAP and recover the catalytic activity due to the cavitation effect. After that, the formed Fe-PDAP produces O_2_ from intracellular H_2_O_2_, enabling efficient ROS production. The SDT process could release TAAs, which could promote dendritic cells (DCs) maturation to educate T cells. 3) MFC could target tumors with a high specificity using the cancer cell membrane due to its superiority in homologous targeting 22, 36, 37. The cell membrane coating can keep the cargoes from eroding and reduce the preliminary leakage of camouflaged agents, which provides significant advantages in our study.^38^, ^39^ Importantly, in combination with ICB therapy (anti-programmed cell death protein-1 antibody, aPD-1), the MFC has demonstrated superb antitumor performance for both primary tumors and distant tumors (**Scheme 1**). Moreover, on-demand O_2_-evolving sonodynamic immunotherapy with MFC induced minimal nonspecific damage to normal tissues, indicating high biocompatibility. Thus, this study provides an intriguing strategy that nanozymes can act as H_2_O_2_ economizers for on-demand O_2_ evolution-assisted robust sonodynamic immunotherapy.

## Methods and Materials

### Reagents and Materials

Fe(III) chloride hexahydrate (FeCl_3_·6H_2_O), [Ru(dpp)_3_]Cl_2_ (RDPP), and SOSG were purchased from Thermo Fisher Scientific (Los Angeles, CA, USA). Diamino pyridine (DAP) was obtained from Adamas-Beta (Shanghai, China). Amplex Red was provided by Aladdin Reagent Co., Ltd. (Shanghai, China). Ce6 was obtained from J&K Chemical Co. aPD-1 was provided by BioxCell (clone: RMP1-14, catalog no. BE0146). The membrane protein extraction kit, phenylmethanesulfonyl fluoride (PMSF), penicillin-streptomycin solution, trypsin, and DCFH-DA were purchased from Beyotime (Shanghai, China). DAPI was obtained from Boster Biological Technologies (Wuhan, China). A standard CCK-8 assay, calcein-AM, and PI were purchased from Dojindo (Japan). All materials were used as received without further purification.

### Cell Lines

4T1 murine breast cancer cells, Panc-1 pancreatic cancer cells, HepG2 hepatocellular carcinoma cells and SKOV3 ovarian cancer cells were provided by Chongqing Medical University. 4T1 cells and SKOV3 cells were maintained in RPMI 1640 complete medium, and Panc-1 cells and HepG2 hepatocellular carcinoma cells were maintained in high-glucose DMEM supplemented with 10% FBS and 1% penicillin-streptomycin in a humidified atmosphere of 5% CO_2_ at 37 °C.

### Animals

Female BALB/c mice (6-8 weeks) were provided by the Animal Center of Chongqing Medical University. All animal studies were carried out under protocols approved by the Animal Ethics Committee of Chongqing Medical University.

### Extraction of Cancer Cell Membranes

Cell membranes were extracted using a membrane protein extraction kit according to the instructions provided by Beyotime Biotechnology. Briefly, cancer cells were seeded in cell culture dishes and scraped off with a cell scraper. The cells were collected by repeated centrifugation (1000 g, 5 min) and distributed into membrane protein extraction reagent pretreated with phenylmethanesulfonyl fluoride (PMSF) in an ice bath for 10-15 min. Afterward, the sample was subjected to repeated freeze-thawing, and centrifugated at 700g for 10min to collect supernatant. Finally, the supernatant was centrifuged (14000 g, 30 min) to obtain the cell membrane fragments.

### Synthesis of Fe-PDAP

Fe-PDAP was synthesized through Fe(III)-mediated oxidative polymerization according to a previously reported method with modifications.^40^ Briefly, FeCl_3_·6H_2_O (10 g) was dissolved in deionized water (200 mL) and sonicated for 10 min. DAP (1.0 g) was added to the mixture and stirred for 24 h at 37 °C to perform polymerization; the obtained mixture was purified with a dialysis bag (MW: 12 kDa) against distilled water overnight. Then, Fe-PDAP was obtained and stored at 4 °C for further use.

### Synthesis of MFC

Ce6 was loaded onto Fe-PDAP by electrostatic adsorption and π-π interactions. In brief, Ce6 (40 mg) were dispersed in Fe-PDAP (60mg) dissolved in methyl alcohol, and magnetically stirred overnight at room temperature. Subsequently, the products were collected by centrifugation (12000 g, 10 min). After that, the obtained cancer cell membranes were mixed with as-prepared Fe-PDAP/Ce6 NPs, and an extrudation method was used to obtain MFC with the aid of a mini extruder (Avastin, Canada). The resulting MFC was further centrifuged to remove the remnants and stored at 4 °C for further use.

### Characterization

TEM images were obtained with a JEM-1400plus (Japan). The particle size and zeta potential measurements were recorded on a Malvern Nano ZS90 (Malvern Instruments, UK). XRD images were taken on a PANalytical X'pert Power System (Spectris Pte. Ltd, Holland). XPS spectra were recorded on an ESCALAB 250 instrument (Thermo Fisher Scientific, USA). UV-Vis absorption spectra were acquired with a UV-Vis-NIR spectrophotometer (UV-3600, Shimadzu, Japan).

### ROS Measurements *In vitro*

TEMP detected the ROS generated by MFC upon exposure to US irradiation. Typically, MFC (20 µg/mL Ce6) was exposed to US irradiation (1.0 MHz, 2 W cm^-2^, 50% duty cycle) for 5 min in the presence of TEMP. The ^1^O_2_ signal was immediately detected by the ESR spectrometer (JEOL-FA200). SOSG was introduced for the quantitative analysis of ^1^O_2_ generation by MFC. Briefly, the SOSG solution was mixed with MFC solution (20 µg/ mL Ce6). Afterward, the mixture was irradiated by US (1.0 MHz, 2 W cm^-2^, 50% duty cycle), and the fluorescence intensity of SOSG was detected with a multimode reader. Intracellular ROS levels were determined with DCFH-DA. DMTU was used to scavenge H_2_O_2,_ and the concentration of DMTU was 20 μM. 4T1 cells were seeded into CLSM-exclusive culture dishes overnight and cocultured with different NPs for 4 h. Then, the cells were washed with PBS and incubated with DCFH-DA (2 × 10^-5^ M) for 30 min, followed by US irradiation (1.0 MHz, 2 W cm^-2^, 50% duty cycle, 1 min). After another 20 min of incubation, the cells were observed by CLSM (λex/λem = 488 nm/530 nm).

### *In vitro* Cytotoxicity Measurements

A standard CCK-8 assay was performed to characterize the cytotoxicity of MFC to 4T1 cells. Typically, the cells were cultured in 96-well plates (5×10^3^ cells per well) overnight to adhere to the wells. Then, different concentrations of MFC were added and incubated for another 24 h, the CCK-8 assay was performed, and a multimode reader was used to determine the results. For the live/dead cell staining assay, 4T1 cells were seeded into CLSM-exclusive culture dishes (1×10^5^ cells per dish) overnight and experienced different treatments. Then the cells were subjected to US irradiation (1.0 MHz, 2 W cm^-2^, 50% duty cycle, 1 min). After that, the cells were stained with calcein-AM and PI for 15 min and observed by CLSM.

### Cellular Uptake Performance of MFC

4T1 cells were seeded in CLSM-exclusive culture dishes overnight and then incubated with different MFCs (cancer cell membranes from different cells) for 1 h. Afterward, the cells were washed with PBS, fixed with 4% paraformaldehyde for 15 min, and incubated with DAPI before observation by CLSM. Excitations: 630 nm for Ce6 and 405 nm for DAPI; emissions: 670 nm for Ce6 and 455 nm for DAPI. Cellular uptake was further evaluated by ICP-MS (Agilent ICPMS 7700) based on the above protocols.

### Determination of Catalytic O_2_ Generation Ability

Different NPs were dispersed in an aqueous solution containing H_2_O_2_ without or with US irradiation (1.0 MHz, 2 W/cm^2^, 50% cycle, 3min). The oxygen concentration was monitored with a dissolved oxygen meter (Mettler Toledo, China). The intracellular production of O_2_ was characterized by an RDPP probe, whose luminescence can be quenched by oxygen. Briefly, 4T1 cells were seeded into CLSM-exclusive culture dishes (1×10^5^ cells per dish) and cultured overnight. The cells were then incubated with RDPP (10 μM) for 4 h, incubated with NPs, and treated with or without US (1.0 MHz, 2 W/cm^2^, 50% cycle,3min). Finally, fluorescence images were captured by CLSM under excitation at 488 nm.

### Evaluation of H_2_O_2_ Consumption Ability

Different NPs (200 μg/mL) were dispersed in an aqueous solution containing H_2_O_2_ without or with US irradiation (1 MHz, 2 W cm^-2^, 50% cycle, 3min). Amplex Red (200 μM) and HRP (0.2 U/ L) were added, and the absorbance of the mixture solution at 590 nm was monitored. 4T1 cells were seeded in a 12-well plate and cultured overnight to evaluate intracellular H_2_O_2_. Then, the cells were coincubated with various NPs for 3 h and exposed to US irradiation (1 MHz, 2 W cm^-2^, 50% cycle, 3min). The cells were collected and broken up with an ultrasonic probe (Sonics & Materials, Inc., USA). Finally, Amplex Red (200 μM) and HRP (0.2 U/ L) were added, and the absorbance at 590 nm was detected.

### Detection of Tumor Hypoxia Status *In vivo*

Intratumoral blood oxygen saturation was evaluated with a Vevo LAZR PAI system (Visual Sonics Inc., Toronto, Canada). Oxygen saturation inside the tumors was measured at the indicated time points after intravenous injection of NPs using Oxyhem mode with excitation wavelengths of 750 and 850 nm. The expression level of HIF-1α was measured by immunofluorescence staining and western blotting at the indicated time points.

### Biodistribution Study of MFC *In vivo*

4T1 tumor-bearing mice were intravenously injected with MFC (3 mg/mL), and fluorescence images were acquired at the indicated time points (1, 2, 4, 6, 8, and 24 h) using a fluorescence live imaging (FLI) system (Xenogen IVIS Spectrum, PerkinElmer, USA). The tumors and major organs (including the heart, liver, spleens, kidneys, and lungs) were imaged *ex vivo* 24 h post-injection.

### *In vivo* Anticancer Therapy

4T1 cell suspensions (1×10^6^) were inoculated into the right mammary fat pad of each mouse as the primary tumor to establish an orthotopic breast cancer model. Four days later, 4T1 cell suspensions (5×10^5^) were inoculated into the left mammary fat pads of mice as distant tumors. When the first tumor reached 50-100 mm^3^ in volume, the mice were randomly distributed into 6 groups and intravenously administered NPs at a dose of 4 mg/mL on days 0, 3, and 6. DMTU was used to scavenge H_2_O_2,_ and the concentration of DMTU was 20 μM. For groups treated with US irradiation, US (1.0 MHz, 2W cm^-2^, 50% duty cycle, 3 min) irradiation was applied to the mice 4 h and 24 h after injection. aPD-1 antibodies at a dose of 100 μg per mouse were intraperitoneally injected on days 1, 4, and 7. Tumor sections of the primary and distant tumors were harvested for H&E and TUNEL staining. The tumor sizes were measured with a Vernier caliper every other day, and the volume was calculated as (width^2^ × length)/2. The body weights of the mice were measured every other day. On day 16, one mouse in each group was sacrificed, and the major organs were collected for H&E staining.

### *Ex vivo* Analysis of Different Groups of Immune Cells

The mice were sacrificed, and then tumor-draining lymph nodes and the distant tumors were harvested on day 8 to explore the underlying immune mechanism of therapy. The tumor-draining lymph nodes were quickly cut into fragments and stained with APC-CD11c (Biolegend, catalog: 117310), FITC-CD80 (Biolegend, catalog: 104705), and PE-CD86 (Biolegend, catalog: 105007). The tumor tissues were quickly cut into fragments, digested with a mixture containing 2% fetal bovine serum (Pan, Germany), 0.02% collagenase IV, and 0.002% DNase I (Solarbio, Beijing, China), ground using the rubber end of a syringe and filtered through a 40 µm nylon cell strainer (NEST, Wuxi, China) to prepare the single-cell suspension. Following removal of the red cells *via* red blood cell lysis buffer and rinsing with PBS, the single-cell suspension was stained with a fluorescently labeled antibody according to the manufacturer's protocols. To analyze CD8^+^ T cells, the cells were labeled with live-dead BV421 (Biolegend, catalog: 423114), CD45-APC-Cy7 (Biolegend, catalog: 103116), CD3-FITC (Biolegend, catalog: 100204), CD4-Percp (Biolegend, catalog: 100431), and CD8-APC (Biolegend, catalog: 100711). The cells were labeled with CD4-FITC (eBioscience, catalog: 11-0041-82), CD25-PE-Cy7 (eBioscience, catalog: 25-0251-82), and Foxp3-PE (eBioscience, catalog: 12-4771-82) to analyze Tregs (CD4^ +^CD25^ +^Foxp3^+^). After different treatments, the mice were sacrificed, and the distant tumors were collected, homogenized, and centrifuged to harvest the supernatant on day 8. Cytokines were analyzed with ELISA kits according to the vendor's instructions (Dakewe Biotech, China).

### *In vivo* Biosafety of MFC

Healthy female Kunming mice (6-8 w) were intravenously administered MFC at a dose of 4 mg /mL. Untreated healthy Kunming mice served as controls (n = 4). The mice were then sacrificed at 1 day, 7 days, 14 days, and 21 days after injection. Blood samples were taken, and the major organs (heart, liver, lung, spleen, and kidney) were harvested for multiple evaluations, including routine blood tests, serum biochemistry analysis, and H&E staining.

### Statistical Analysis

All data are presented as the mean ± standard deviation (SD), and statistical analysis of the data was performed by Student's t-test and one-way ANOVA. *p < 0.05, **p < 0.01, ***p < 0.001.

## Results and Discussion

### Synthesis and Characterization of MFC

**Figure 1:**
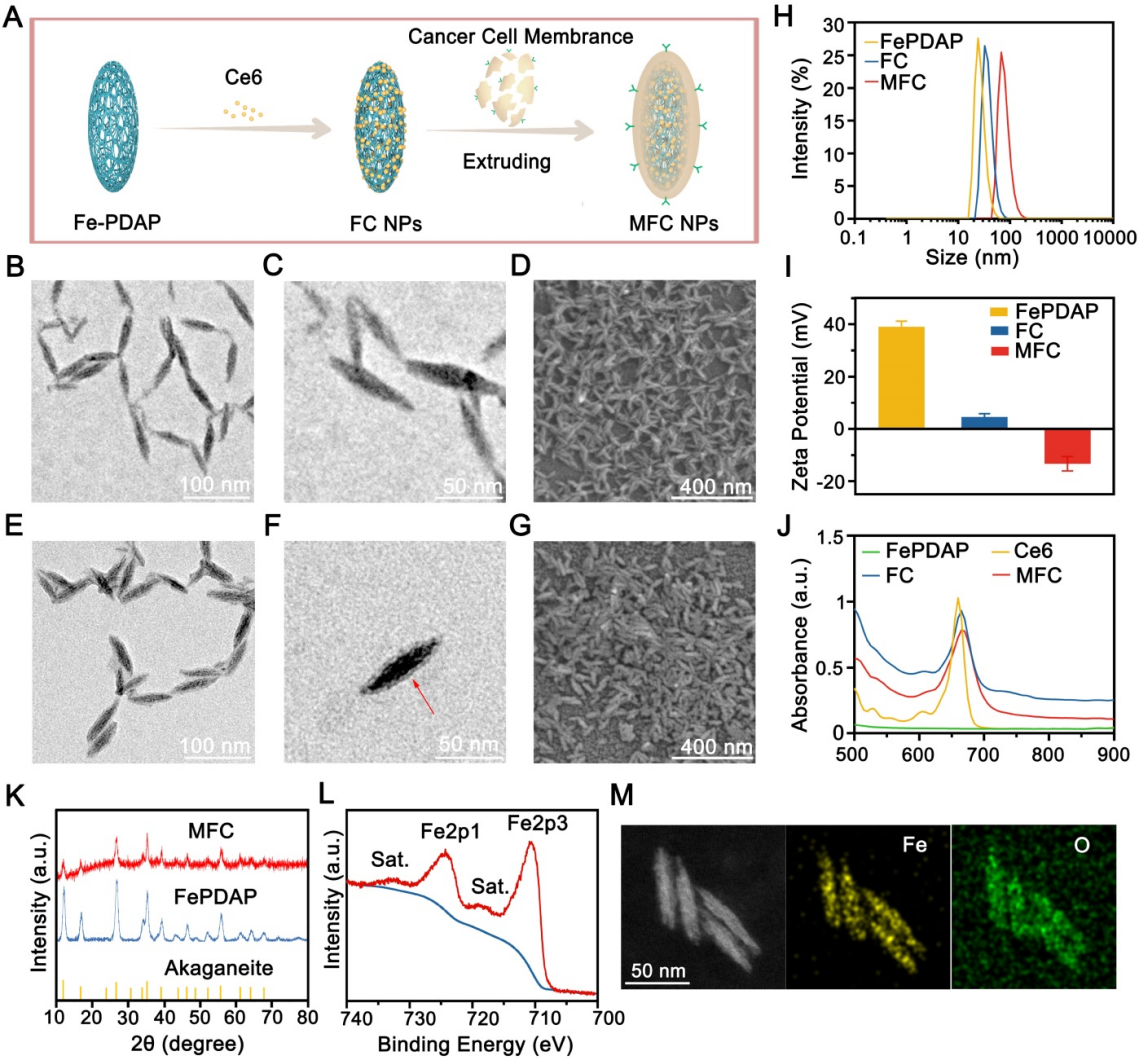
MFC characterization. (A) Schematic illustration of the synthetic procedures for engineered MFCs. (B-D) TEM and SEM images of Fe-PDAP. (E-G) TEM and SEM images of MFC. (H) Size distribution of Fe-PDAP, FC, and MFC. (I) Zeta potentials of Fe-PDAP, FC, and MFC. (J) UV/vis absorption spectra of Fe-PDAP, Ce6, FC, and MFC. (K) XRD patterns of MFC and Fe-PDAP. (L) XPS high-resolution Fe2p spectrum of MFC. (M) Elemental mappings of MFC.

Fe-PDAP with catalase-like activity was prepared using a Fe(III) mediated oxidative polymerization strategy (**Figure 1A**). Transmission electron microscopy (TEM) images and scanning electron microscopy (SEM) images revealed fusiform-like shaped Fe-PDAP with a uniform morphology (**Figure 1B**-**D**). The obtained Fe-PDAP (polydispersity index, PDI:0.234) showed a narrow hydrodynamic size distribution of approximately 40 nm and a positive charge potential of +39 mV (**Figure 1H**-**I**). Then, we placed the Fe-PDAP aqueous solution in a dialysis bag, and the solution was replaced with methanol at room temperature for subsequent loading of Ce6. Ce6 was loaded onto Fe-PDAP through hydrophobic and π-π interactions after stirring in a methanolic solution to prepare Fe-PDAP/Ce6 (FC). After loading, the size distribution of FC (PDI:0.215) increased to approximately 60 nm (**Figure 1H**), and its zeta potential reversed to +4.59 mV (**Figure 1I**), which indicates the successful loading of Ce6. However, the loading agents can hardly be observed under TEM, most likely because the layer has a low electron density (**Figure S1**). The cracked cell membranes extracted from the 4T1 cells were used as the shells of FC to prepare cancer cell membrane-coated FC (MFC, PDI:0.18) through a facile coextrusion method. Compared to naked FC, MFC showed a prominent core-shell structure with a uniform lipid bilayer on the shell, indicating the successful cloaking of the cancer membrane (**Figure 1E**-**G**). Moreover, coating cancer cell membranes to FC led to a mild increase in hydrodynamic size and a positive-to-negative reversal of zeta potential (**Figure 1H**-**I**). In addition, compared to that of free Ce6, the UV-Vis absorption spectra of MFC showed a slight red-shift phenomenon from 660 to 665 nm, which revealed electrostatic adsorption and π-π interactions between Ce6 and Fe-PDAP (**Figure 1J**). According to the UV-Vis analysis, we calculated the encapsulation efficiency of Ce6 as 72%, and the loading capacity of Ce6 was 48%. (**Figure S2**). The color discrepancy between Fe-PDAP and MFC further confirmed the successful loading of Ce6 (**Figure S3**). We have also examined the stability of MFC in different mediums. The results showed that the size of MFC remained unchanged in H_2_O and 1640 containing 10% FBS wihtin 15 days (**Figure S4**), indicating the high stability of MFC. No noticeable difference was found between the powder X-ray diffraction (XRD) patterns of Fe-PDAP and MFC, suggesting a negligible effect of the loading process on the crystal structure of Fe-PDAP (**Figure 1K**). The peak of Fe-PDAP was recognized with akaganeite according to the standard XRD card. The typical binding energy peaks of Fe^1/2^ and Fe^3/2^ in the X-ray photoelectron spectroscopy (XPS) showed that approximately 100% Fe in MFC was in the chemical state of Fe(III) (**Figure 1L, Figure S5**). Fe(III) could reduce the toxic Fenton reaction and improve the biosafety of the MFC after exposure to an acidic environment. Elemental mapping analysis confirmed the existence of Fe in the MFC (**Figure 1M**), and we obtained an Fe content of 3.93 wt% according to inductively coupled plasma mass spectrometry (ICP-MS) results.

### *In vitro* On-demand Catalase Activity of the NPs and the H_2_O_2_ content during treatment

**Figure 2:**
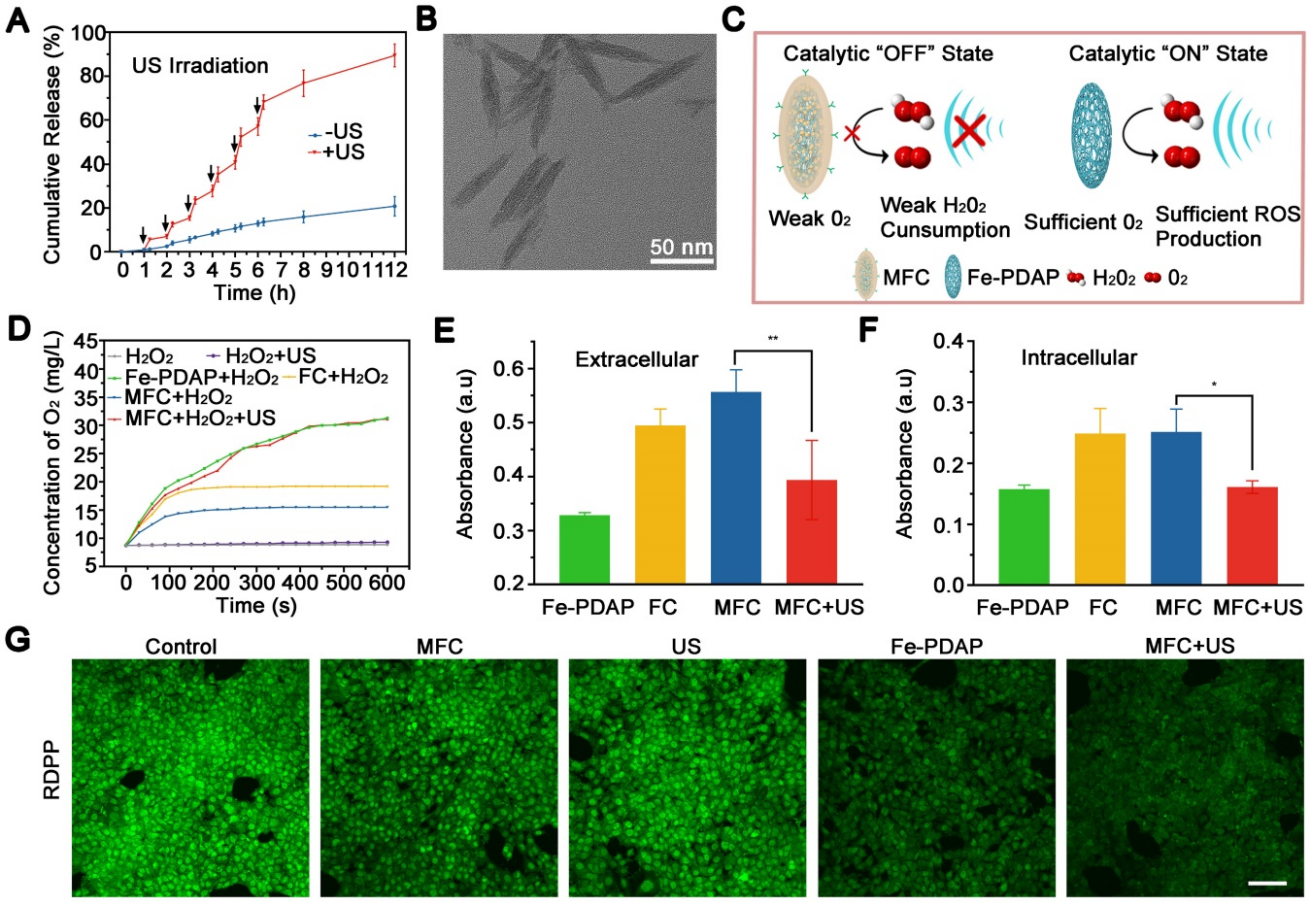
*In vitro* on demand catalase-like activity of the NPs and the H_2_O_2_ content during treatment. (A) Ce6 controlled release of MFC triggered by US irradiation. (B) TEM of MFC after US exposure. (C) Schematic illustration of the therapeutic mechanism in the presence of US-detachable catalase-like nanozymes. (D) The production of O_2_ by different NPs with or without US irradiation. (E) Extracellular H_2_O_2_ content after different treatments measured by Amplex Red. (F) Intracellular H_2_O_2_ content after different treatments measured by Amplex Red. (G) Confocal laser scanning microscopy (CLSM) images of RDPP in 4T1 cells after different treatments. Scale bar: 100 μm.

Under US irradiation, MFC showed an evident US-responsive burst of Ce6 release, with a total Ce6 leakage of approximately 80% after repeated US irradiation, which could be ascribed to US-induced MFC disintegration (**Figure 2A**). In comparison, the Ce6 release rate was relatively low (~20%) without US treatment, indicating the excellent chemical stability of MFC. The morphology of US-treated MFC was also tested, and the core-shell structure was broken under TEM observation (**Figure 2B**), which could be ascribed to acoustic radiation forces and cavitation effect induced disintegration.^41^, ^42^ US-treated MFC showed a decrease in hydrodynamic size and a negative-to-positive reversal of zeta potential, also confirming the breakage of the core-shell structure (**Figure S6**). As such, both the the removal of cell membrane or the detachment of Ce6 was achieved and the exposure of Fe-PDAP can initiate catalytic effect.

Since H_2_O_2_ content has a powerful influence on O_2_ generation during SDT treatment, it is essential to confirm the catalytic activity changes upon US irradiation under MFC treatment (**Figure 2C**). We evaluated the catalase activity of Fe-PDAP with a portable dissolved oxygen meter after the addition of H_2_O_2_. As expected, a noticeable increase in the concentration of dissolved O_2_ suggested that Fe-PDAP could decompose H_2_O_2_ (**Figure S7**). However, the decoration process substantially blocked the catalytic effect of Fe-PDAP, and minimal O_2_ generation was detected, indicating that the decoration process reduced the consumption of H_2_O_2_ (**Figure 2D**). The US-detachable properties of MFC inspired us to test the influence of US on the catalytic effect. It was fascinating to find that US treatment could significantly enhance the catalytic effect of MFC, and recover to reach the ability of naked Fe-PDAP (**Figure S8**). Next, we employed Amplex Red to test the H_2_O_2_ content both extracellularly and intracellularly under different treatments.^43^, ^44^ After the H_2_O_2_ solution was treated with Fe-PDAP, noticeable H_2_O_2_ depletion was observed compared to the control groups (MFC or FC alone), confirming that the H_2_O_2_ depletion capability was reduced by the decoration process (**Figure 2E**). In addition, in the group treated with MFC+US, the H_2_O_2_ content was comparable to that of the Fe-PDAP alone group. These results revealed that MFC could reduce H_2_O_2_ consumption, and US could achieve on-demand catalysis to enhance the O_2_ supply during SDT. Similarly, we evaluated intracellular H_2_O_2_ consumption under different treatments. There was reduced H_2_O_2_ consumption in the MFC group, and the groups treated with MFC+US showed H_2_O_2_ consumption similar to that of the Fe-PDAP group (**Figure 2F**). These phenomena enable on-demand depletion of H_2_O_2_ and provide an excellent method for supplying O_2_ to overcome tumor hypoxia. The effectiveness of MFC+US in achieving on-demand O_2_ production was further investigated in 4T1 cells using an RDPP probe, whose fluorescence can be quenched by intracellular O_2_.^45^ The results showed that the 4T1 cells incubated with naked Fe-PDAP or MFC+US showed notably quenched fluorescence compared with the other groups. The drastic H_2_O_2_ decrease in the MFC+US group supports our hypothesis that US-treated MFC could improve hypoxia relief in tumor cells by on-demand oxygen supply (**Figure 2G, Figure S9**).

To explore the limited time for the release of Fe-PDAP, MFC and the main composition of MFC (including Fe-PDAP, Ce6, and cancer cell membrane) were co-incubated with 4T1 cells, and different ultrasonic irradiation time was performed. The MFC treated groups were set as the on-demand O_2_ supply group, while the solution treated with Fe-PDAP, Ce6, and cancer cell membrane was set as the currently prevalent O_2_ supply approach group. To confirm the content of Ce6 was equivalent in the two groups, CLSM was carried out to observe the fluorescence intensity. A 2 times amount of Ce6 in the currently prevalent O_2_ supply approach group (including Fe-PDAP, Ce6, and cancer cell membrane) was added to achieve the same fluorescence intensity between the two groups. After 4 h of incubation, a higher level of ROS generation in the MFC groups was observed in all the observation times from the 30s to 90s (**Figure S10**), indicating a higher ROS generation capability of MFC with the assistance of ultrasound. Therefore, we believe the on-demand oxygen supply method for enhancing sonodynamic therapy is highly efficient.

### *In vitro* Cytotoxicity Effects of On-Demand Sonodynamic Therapy

**Figure 3:**
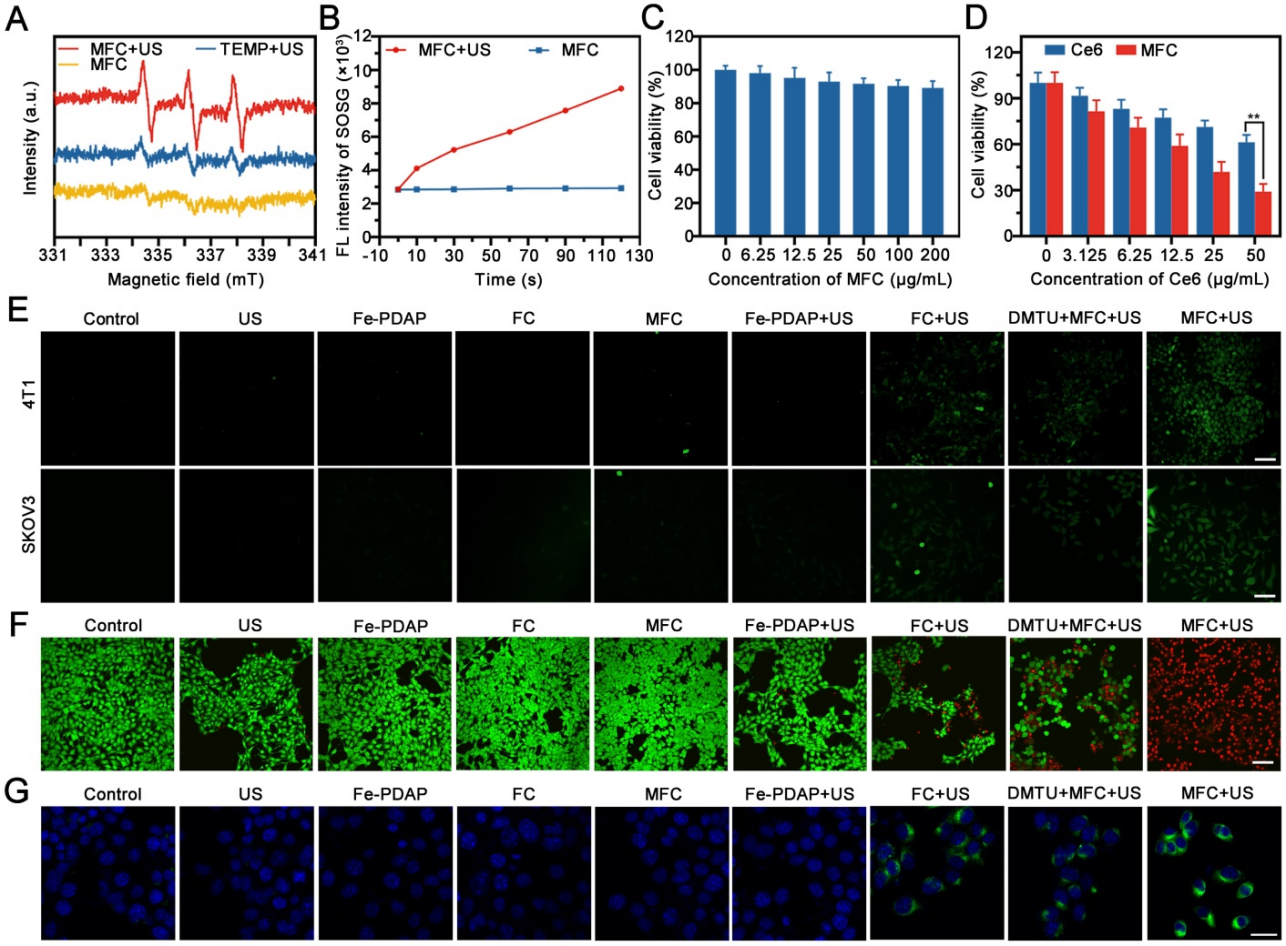
*In vitro* cytotoxicity assay and therapeutic effects. (A) ESR spectra of MFC with or without US irradiation. (B) Quantitative analysis of ROS production by MFC with or without US irradiation using SOSG as a probe. (C) Cell viability of 4T1 cells after incubation with MFC for 24 h. (D) Cell viability of 4T1 cells after treatment with Ce6 or MFC at various concentrations of Ce6 after exposure to US irradiation. (E) CLSM images of 4T1 cells and SKOV3 cells stained with DCFH-DA after different treatments among different groups. Scale bar: 100 μm. (F) CLSM images of 4T1 cells costained with PI and calcein-AM after different treatments. Scale bar: 100 μm. (G) Representative CLSM images showing CRT exposure on 4T1 tumor cells after different treatments. scale bar = 25 μm.

We first evaluated the therapeutic activity of MFC *in vitro* by testing US irradiation-induced ROS generation with electron spin resonance (ESR) spectroscopy. The results showed that the US could efficiently excite MFC to generate ROS (**Figure 3A**). Moreover, the singlet oxygen sensor green (SOSG), which possesses high specificity for ^1^O_2_, was used as a ROS probe to detect the generation of ^1^O_2_ under US irradiation. All tested time points showed fluorescence enhancement after US irradiation and a time-dependent signal intensity enhancement (**Figure 3B**). Next, we studied the *in vitro* biosafety of MFC using a standard cell counting kit-8 (CCK-8) assay. Negligible cytotoxicity was observed toward 4T1 cells after incubation for 24 h (**Figure 3C**). In addition, the hemolysis assay results showed that the MFC has high biosafety for *in vivo* applications (**Figure S11**). The *in vitro* cytotoxicity of MFC or Ce6 irradiated by US activation was also evaluated by CCK8 assay. The cell viability of all treatment groups decreased with increasing Ce6 concentration, indicating that the therapeutic effect was concentration-dependent (**Figure 3D**). The harmful ROS in tumor cells is considered the most representative therapeutic unit to induce intracellular oxidative stress and cell death. Therefore, the intracellular ROS levels after incubation with MFC were detected using 2',7'-dichlorofluorescein diacetate (DCFH-DA), a ROS indicator that can be converted to 2',7'-dichlorofluorescein (DCF) and emits green fluorescence under CLSM observation. 1, 3-Dimethylthiourea (DMTU) was used to scavenge H_2_O_2_ and mimic a microenvironment lacking H_2_O_2_. [46]There was a significant fluorescence signal in the MFC+US group (**Figure 3E, Figure S12**). In contrast, much weaker fluorescence was observed in the DMTU+MFC+US group, indicating that MFC could initiate more effective ROS generation and H_2_O_2_ plays an essential role in enhancing SDT. Additionally, to intuitively observe the distribution of live and dead 4T1 cells, the fluorescent dyes calcein-AM (green) and propidium iodide (PI) (red) were used.^47^ The results showed that 4T1 cells could be killed more efficiently in the MFC+US group than the other groups after 4 h of incubation, demonstrating the effectiveness of MFC in on-demand producing intracellular ROS for tumor therapy (**Figure 3F, Figure S13**). To make the evaluation of on-demand sonodynamic therapeutic efficacy more persuasive, we have built another tumor cells (SKOV3 cells) for further evaluation. The therapeutic efficacy in SKOV3 cells was highly consistent with the results presented in 4T1 cells. Therefore, we believe that our on-demand sonodynamic therapy has the potential to be extended to other tumors. Immunogenic cell death (ICD) has been regarded as a requirement for effective immunotherapy. Calreticulin (CRT) is a significant biomarker of ICD. It has been reported that the intracellular oxidative stress caused by SDT could potentially trigger CRT expression on the surface of tumor cells, increase the immunogenicity of tumors and mobilize the immune system. Therefore, the expression of cell surface CRT induced by MFC-enhanced SDT was evaluated by CLSM *in vitro*. The MFC+US group showed the highest CRT expression on the surface of tumor cells, which could be ascribed to massive intracellular ROS generation (**Figure 3G**). The results indicated that MFC-enhanced SDT could efficiently induce ICD and activate the immune system.

### Tumor Hypoxia Status Detection *In vivo*

**Figure 4:**
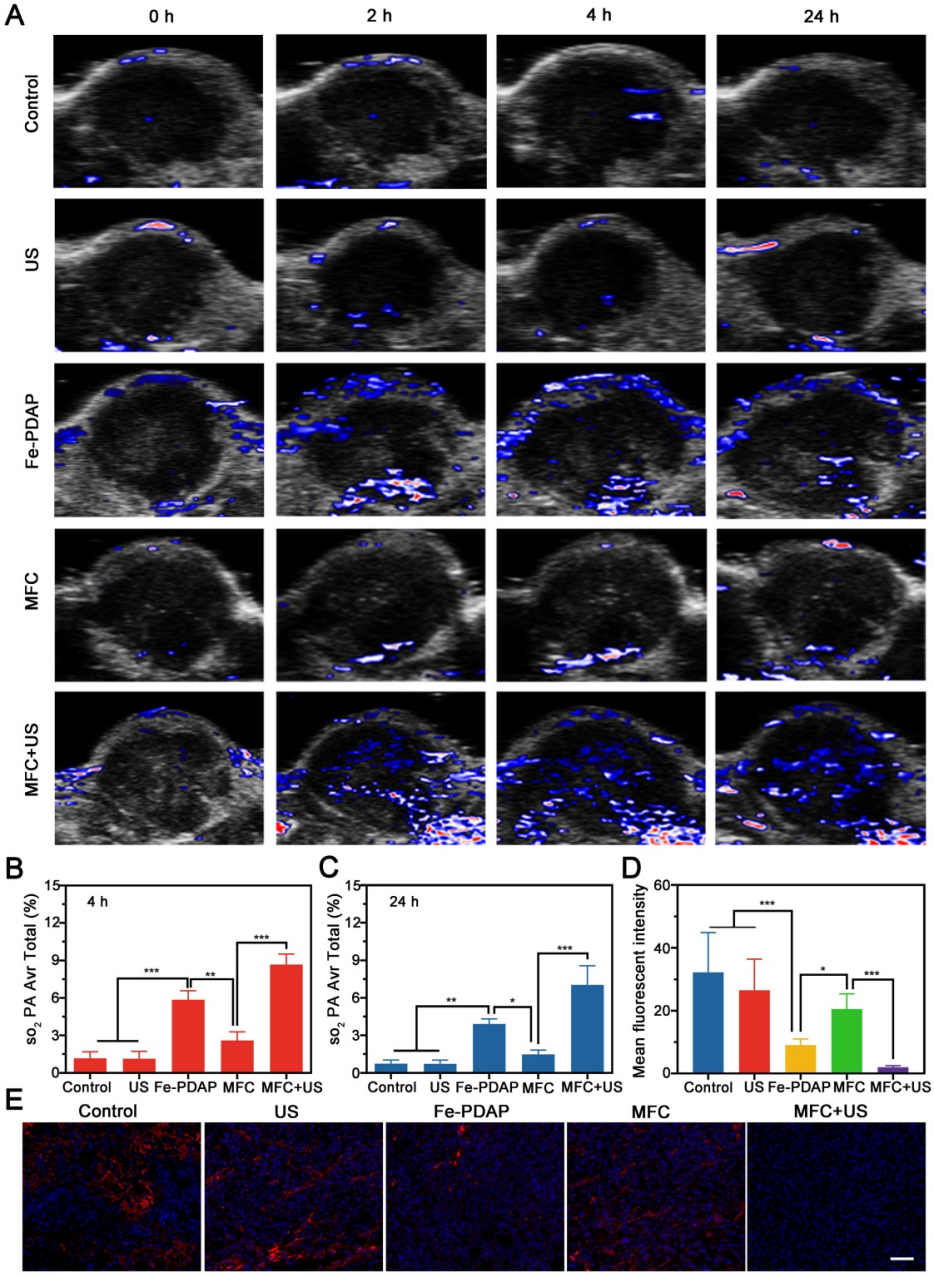
*In vivo* hypoxia relief after different treatments. (A) Representative PA images of tumor sites in 4T1 tumor-bearing mice after various treatments and the corresponding signal intensity values at (B) 4 h and (C) 24 h. (D-E) Representative immunofluorescence images of tumor sections stained with a HIF-1α after different treatments and the corresponding signal intensity values. Scale bar: 50 μm.

The *in vivo* catalytic behavior of MFC was systematically evaluated using photoacoustic imaging (PAI). This noninvasive method can discriminate oxygenated hemoglobin from deoxygenated hemoglobin according to the absorbance spectrum.^48^ As shown in **Figure 4A**, minimal oxyhemoglobin signal intensities were observed in the control and US-only groups, indicating that the US alone could not induce O_2_ generation in the tumor microenvironment. In contrast, the Fe-PDAP group showed enhanced oxyhemoglobin signal intensities within the tumor region, confirming that the presence of Fe-PDAP could improve oxygenation in hypoxic tumors *via* the decomposition of excess H_2_O_2_. Notably, the oxyhemoglobin signal intensities of the MFC group were significantly lower than those of the Fe-PDAP alone group, indicating that the catalytic activity was remarkably inhibited by the surface-modified structure (**Figure 4B**-**C**). However, US treatment led to significantly increased MFC catalytic activity, producing the highest blood oxygen saturation among the groups. The above results suggested that decoration on the Fe-PDAP surface could avoid additional H_2_O_2_ consumption and that US irradiation could switch on the enzymatic activity of Fe-PDAP to afford robust SDT through on-demand O_2_ supply.

To further verify the relief of tumor hypoxia induced by MFC+US, tumors were collected 24 h after various treatments for immunofluorescence staining of HIF-1α. As shown in **Figures 4D** and **4E**, the HIF-1α signals showed a reduction in the tumors injected with Fe-PDAP. Comparatively, the tumor tissue treated with MFC+US showed a more weakened expression of HIF-1α during the observation period. These results indicated that decoration of Fe-PDAP could achieve on-demand oxygen production with US assistance and indeed lead to robust tumor hypoxia relief. Furthermore, the expression of HIF-1α was also investigated by western blot analysis. The results showed that the expression of HIF-1α/β-actin was significantly reduced in the MFC+US group compared to the MFC-only group and the control group (**Figure S14**), indicating that US-treated MFC provides significant hypoxia alleviation for O_2_-assisted SDT. Moreover, the expression of HIF-1α/β-actin in the MFC-only group was almost the same as that in the control group, suggesting that the catalytic activity of MFC was almost turned off. Therefore, the remarkable SDT effect and on-demand tumor oxygenation enhancement observed with MFC could ensure potent tumor inhibition.

### Homologous Targeting Properties of MFC

**Figure 5:**
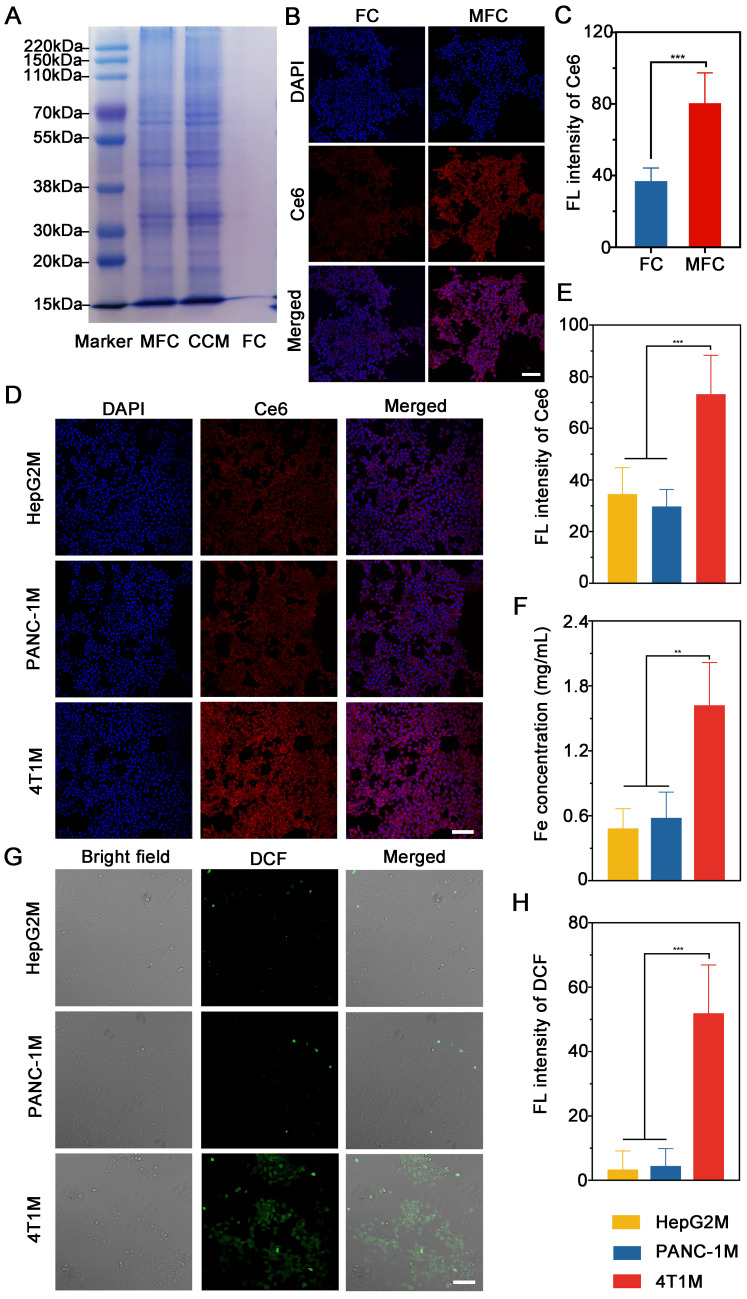
*In vitro* active targeting effects of MFC. (A) SDS-PAGE analysis of MFC, cancer cell membranes, and FC. (B-C) CLSM images of the 4T1 cells incubated with FC and MFC, and the corresponding quantitative fluorescence intensity of Ce6. Scale bar: 100 μm. (D-E) CLSM images of 4T1 cells treated with FC cloaked with different cancer cell membranes and the corresponding quantitative fluorescence intensity of Ce6. Scale bar: 100 μm. (F) Intracellular Fe content in 4T1 cells after treatment with FC cloaked with different cancer cell membranes. (G-H) CLSM images of 4T1 cells stained with DCFH-DA after treatment with FC cloaked with different cancer cell membranes and the corresponding fluorescence intensity of DCF. Scale bar: 100 μm.

Functionalized adhesion proteins on the cancer cell membrane have been considered critical for achieving specific homologous targeting ability. To investigate the homotypic targeting effects of the cancer cell membrane, we first evaluated the overall protein components of MFC by sodium dodecyl sulfate-polyacrylamide gel electrophoresis (SDS-PAGE). The results showed that MFC retained almost all the proteins from the original 4T1 cell membrane, indicating minimal protein loss during the decoration process (**Figure 5A**). To assess the homologous targeting effects of the MFC, the cellular uptake of FC and MFC was conducted on 4T1 cells and observed by CLSM. The MFC group showed stronger Ce6 fluorescence in the cytoplasm of 4T1 cells than that of FC group (**Figure 5B-C**). To further evaluate the homologous targeting effects of the MFC, the prepared FC was decorated with different cell membranes, and CLSM and ICP-MS were used to assess internalization by 4T1 cells. FC cloaked with PANC-1 pancreatic cancer cell membranes (PANC-1M), HepG2 hepatocellular carcinoma cell membranes (HepG2M), or 4T1 breast cancer cell membranes (4T1M) were incubated with 4T1 cells for 1 h. The results showed obvious Ce6 fluorescence in the 4T1M group in the cytoplasm of 4T1 cells, whereas much weaker fluorescence was found in both the PANC-1M and HepG2M groups (**Figure 5D**-**E**). The marked differences in red fluorescence among these treatments demonstrated that the 4T1 cancer cell membranes exhibited the highest affinity for homologous cells because the as-prepared MFC displayed the same cell adhesion molecules as the original cancer cells. The superior homologous targeting properties of MFC were also confirmed by ICP-MS, in which the cells treated with the 4T1M-originated MFC had an average Fe content of 1.6 mg/mL, which was almost 3 times higher than that of the PANC-1M- or HepG2M-originated MFCs (**Figure 5F**).

After validating the homologous targeting properties of MFC NPs on 4T1 cells, we further studied their functional role in antitumor SDT separately using heterologous membranes as controls. Contrary to decoration with PANC-1M or HepG2M, 4T1M decoration showed significantly enhanced DCF fluorescence with US irradiation under the same conditions. These findings further validated the effectiveness of tumor-specific therapy *in vitro* for further sonodynamic immunotherapy (**Figure 5G**-**H**). Thus, the results above supported our conclusion that the MFC originating from 4T1M could effectively be enriched in 4T1 cells *via* homologous targeting mechanisms, further demonstrating the particularly strong binding and/or uptake efficiency of NPs by their original cells.

### *In vivo* Evaluation of On-demand Oxygen Production-Assisted Sonodynamic Immunotherapy

FL imaging was used to evaluate tumor accumulation and biodistribution of MFC. MFC was intravenously injected into mice bearing orthotopic 4T1 tumors and observed by an *in vivo* imaging system. The fluorescence signal in the tumor was detected 1 h after injection of MFC, and a high accumulation of MFC in the tumor was recorded, reaching a peak at 4 h post-injection and gradually decreasing after 4 h of observation, demonstrating an optimized time window for SDT (**Figure S15**). Even up to 24 h after injection, the tumor tissues retained high uptake of MFC, as evidenced by the fluorescence signal (**Figure S16**). Therefore, the tumor-bearing mice were subjected to US irradiation at 4 h and 24 h after intravenous injection of the MFC.

**Figure 6:**
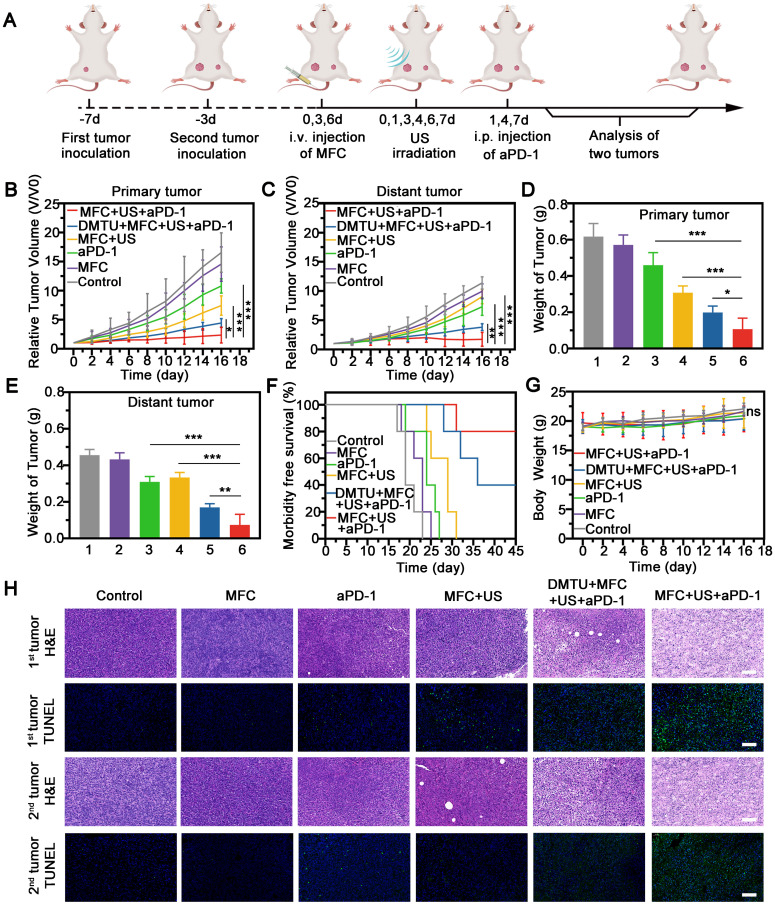
Therapeutic efficacy of MFC combined with aPD-1-mediated on-demand sonodynamic immunotherapy. (A) Schematic illustration of MFC-mediated SDT plus aPD-1 to suppress the growth of tumors at the primary or distant site in a tumor-bearing mouse model. (B-C) Growth curves of primary tumors and distant tumors. (D-E) Tumor weights of primary and distant tumors after different treatments.(1:Control, 2:MFC, 3:aPD-1, 4:MFC+US, 5:DMTU+MFC+US+ aPD-1, 6:MFC+US+aPD-1) (F) Morbidity-free survival of mice after various treatments. (G) Time-dependent body weight curves of the 4T1 tumor-bearing mice after different treatments. (H) H&E staining and TUNEL staining of primary and distant tumors. Scale bar: 100 μm.

Next, we investigated the potential of MFC for sonodynamic immunotherapy with a 4T1 tumor model, as shown in a schematic illustration (**Figure 6A**). Briefly, a murine orthotopic tumor model was established by injecting 4T1 cells into the right mammary fat pad of female BALB/c mice (the primary tumor). After 4 days of incubation, a second tumor was injected into the left side of the mammary fat pad (as the distant tumor). The mice with 4T1 orthotopic tumors were divided into six groups and treated with PBS, MFC, aPD-1, MFC+US, DMTU+MFC+US+aPD-1, or MFC+US+aPD-1. aPD-1 at a dose of 100 µg/mouse was injected intraperitoneally on the 1^st^, 4^th^, and 7^th^ days. The lack of H_2_O_2_ content in the tumor microenvironment is often accompanied by resistance to sonodynamic therapy due to their ability to produce O_2_. Therefore, DMTU was intratumorally injected into the primary tumor 24 h before treatment to scavenge H_2_O_2_ and mimic a tumor microenvironment lacking H_2_O_2_. We found that MFC+US could inhibit the growth of the primary tumors (**Figure 6B, Figure S17**). However, sonodynamic therapy alone failed to inhibit the distant tumors (**Figure 6C, Figure S17**). After combination with aPD-1, MFC-reinforced SDT+aPD-1 more effectively suppressed both primary and distant tumors (**Figure 6D-6E**), further demonstrating the excellent tumor inhibition efficacy of sonodynamic immunotherapy. Comparatively, DMTU+MFC+US+aPD-1 only had a moderate effect on tumor inhibition, which consolidated the therapeutic efficacy of MFC by on-demand O_2_ production augmented sonodynamic immunotherapy. Survival analysis showed that 80% percent of mice exposed by MFC-reinforced SDT+aPD-1 survived to day 45, more than 2-fold higher than that of other groups (**Figure 6F**). Hematoxylin-eosin (H&E) and TdT-mediated dUTP nick-end labeling (TUNEL) staining of the tumor sections further revealed the most significant apoptosis and necrosis of tumors in the MFC+US+aPD-1 group (**Figure 6H**). Therefore, we reasonably believe that the on-demand O_2_ production augmented sonodynamic therapy combined with immune checkpoint blockade was likely more efficient than traditional successive administration of sonodynamic immunotherapy.

Regarding safety, no noticeable weight or temperature changes were recorded throughout the observation period among the groups (**Figure 6G, Figure S18**). H&E staining of the major organs (including the heart, liver, spleen, lungs, and kidneys) revealed negligible toxicity among the six groups (**Figure S19**). The results indicated that the therapeutic dose in our work was well tolerated. The biocompatibility of the therapeutic process was further assessed in healthy Kunming mice by routine blood tests and blood biochemical analysis, and no obvious differences were observed among the groups at 21 days posttreatment (**Figure S20**). In addition, H&E staining of the major organs (including the heart, liver, spleen, lungs, and kidneys) displayed no obvious pathological damage or inflammatory lesions during our observation time (**Figure S21**). The ICP results showed that the MFC has a half-time time of 8.69 h, indicating the high biosafety of MFC with fast elimination capability in blood circulation (**Figure S22**). Overall, MFC showed remarkable antitumor effects with minimal systemic toxicity after sonodynamic immunotherapy.

### Antitumor Immune Mechanisms

**Figure 7:**
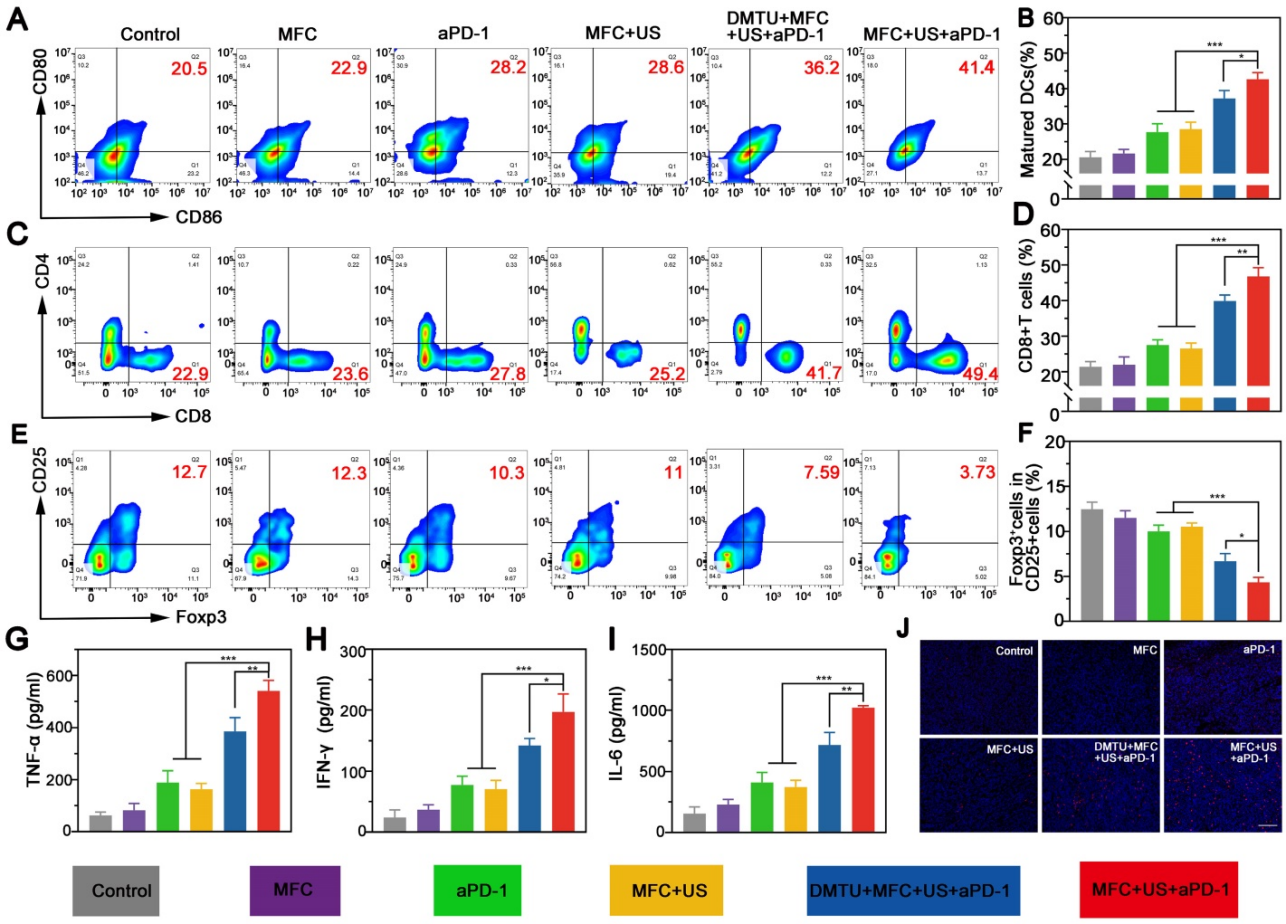
The immune mechanistic study. (A) Flow cytometry assay of matured DCs (CD80 ^+^ CD86^ +^ gated on CD11c^+^) in tumor-draining lymph nodes of mice after different treatments and (B) corresponding quantitative data. (C) Representative flow cytometry plots of CD8^+^ T cells (gated on CD3^+^ cells) and (D) corresponding quantitative data in distant tumors after different treatments. (E) Representative flow cytometry plots of Tregs (CD4^+^CD25^+^Foxp3^+^) (gated on CD4^+^ cells) (F) corresponding quantitative data in distant tumors after different treatments. (G,H,I) Cytokine levels in distant tumor tissues after different treatments. (J) Immunofluorescence staining of CD8^+^ T cell infiltration in distant tumors after various treatments. Scale bar: 100 μm.

Sonodynamic therapy (SDT) can trigger immunogenic cell death (ICD) with release of tumor-associated antigens (TAAs). The released TAAs could facilitate the maturation of dendritic cells (DCs) in the tumor-draining lymph nodes and present TAAs to activate effector T cells. To explore the underlying mechanism of the tumor-specific immunological effect evoked by MFC+US+aPD-1, tumor-draining lymph nodes were harvested on the 8^th^ day and analyzed by flow cytometry. The results showed MFC+US+aPD-1 could effectively elevate the percentage of mature DCs from 20.5% (Control) to 41.4% (MFC+US+aPD-1) (**Figure 7A**-**B**). Moreover, DMTU could significantly reduce the maturation of DCs, further indicating the importance of H_2_O_2_ in MFC-based sonodynamic immunotherapy.

CD8^+^ T cells (CD3^+^CD4^-^CD8^+^), known as CTLs, can kill tumor cells directly and are vital in the antitumor immune response.^49^, ^50^ Therefore, CD8^+^ T cell infiltration in distant tumors at the 8^th^ day was examined by flow cytometry. The results showed that the percentage of CD8^+^ T cells in the MFC+US+aPD-1 group was 49.4%, significantly increasing (by nearly 2.1-fold) compared to the control group (**Figure 7C**-**D**). Moreover, the proportion of CD8^+^ T cells in the MFC+US+aPD-1 group was higher than those in both the MFC+US group and the aPD-1 group, indicating that the therapeutic outcome was markedly better than that of either SDT or immunotherapy alone. Moreover, DMTU could significantly reduce the infiltration of CD8^+^ T cells, further indicating the importance of H_2_O_2_ in sonodynamic immunotherapy and the on-demand O_2_ supply could enhance the efficacy of sonodynamic immunotherapy. Unlike CD8^+^ T cells, regulatory T cells (Tregs, CD4^+^CD25^+^Foxp3^+^) can protect tumor cells to avoid attack from the immune system and suppress the antitumor immune response.^51^, ^52^ Therefore, the distant tumor was collected at the 8^th^ day and costained with CD4, CD25, and Foxp3 for further analysis. The results showed that the percentage of Tregs in the MFC+US+aPD-1 group was much lower than that in the other groups (**Figure 7E**-**F)**, demonstrating significant attenuation of the immunosuppressive environment of the tumor. Secreted cytokines, including tumor necrosis factor-α (TNF-α), interferon-γ (IFN-γ) and interleukin-6 (IL-6), are also important biomarkers that can indicate the potency of the immune response. Based on this, we further explored the levels of cytokines in distant tumors after various treatments on the 8^th^ day. There was a significant increase in the production of TNF-α, IFN-γ, and IL-6 in the MFC+US+aPD-1 group compared to the control, monotherapy groups, and DMTU+MFC+US+aPD-1 group (**Figure 7G**-**7I**), verifying the strong antitumor immune potency induced by MFC-based SDT plus aPD-1. In addition, the infiltration of CD8^+^ T cells into distant tumors was evaluated by immunofluorescence staining. As shown in **Figure 7j**, the infiltration of CD8^+^ T cells was significantly increased in the MFC+US+aPD-1 group, indicating that MFC+US+aPD-1 could effectively recruit T cells to initiate a robust immune response.

## Conclusions

In conclusion, we developed the H_2_O_2_ economizer MFC to overcome the limitations of hypoxia in sonodynamic immunotherapy. By taking full advantage of the ultrasonic cavitation effect, we endowed MFC with US-responsive disintegration characteristics. The catalytic activity of Fe-PDAP was greatly reduced after attachments of Ce6 and cancer cell membranes, which successfully achieved on-demand O_2_ production without the additional consumption of H_2_O_2_. By employing a biomimetic engineering strategy with cancer cell membranes, we addressed the premature leakage issue and increased tumor-site accumulation of MFC. We confirmed the hypoxia relief capability of MFC+US in tumor cells, while MFC alone could not efficiently alleviate tumor hypoxia. Upon US irradiation, the accumulated MFC disintegrated into Fe-PDAP to further improve tumor oxygenation in an on-demand manner. In addition, systemic administration of MFC+US combined with aPD-1 showed not only superior efficacy in combatting both primary, but also distant tumors. This study established a “broadening source of H_2_O_2_” strategy for the reversal of hypoxia during O_2_-dependent sonodynamic immunotherapy. It also provided a strategy between O_2_-enhanced sonodynamic therapy and immunotherapy for eradicating tumors.

## Supplementary Material

Supplementary figures.

## Abbreviations

Fe-PDAP: Fe-doped polydiaminopyridine
SDT: Sonodynamic therapy
aPD-1: anti-programmed cell death protein-1 antibody
ROS: reactive oxygen species
ICD: immunogenic cell death
TAAs: tumor-associated antigens
TME: tumor microenvironment
H_2_O_2_: hydrogen peroxide
Ce6: chlorin e6
DCs: dendritic cells
TEM: transmission electron microscope
SEM: scanning electron microscope
PDI: polydispersity index
ICP-MS: inductively coupled plasma mass spectrometry
PMSF: phenylmethanesulfonyl fluoride
ESR: electron spin resonance
SOSG: singlet oxygen sensor green
CCK-8: cell counting kit-8
DMTU: 1, 3-Dimethylthiourea
CLSM: confocal laser scanning microscope
PAI: photoacoustic imaging
SDS-PAGE: sodium dodecyl sulfate-polyacrylamide gel electrophoresis
HE: hematoxylin & eosin
TUNEL: terminal deoxynucleotidyl transferase-mediated dUTP-biotin nick and labeling
