# Supplementary material for: A hydrogen peroxide economizer for on-demand oxygen production-assisted robust sonodynamic immunotherapy
Source: Theranostics. 2022 Jan 1;12(1):59–75. doi: 10.7150/thno.64862 (PMC8690934; doi:10.7150/thno.64862)
Supplement: Supplementary file 1 — Supplementary figures. [file thnov12p0059s1.pdf]

Supporting information

A hydrogen peroxide economizer for on-demand oxygen production-assisted robust sonodynamic immunotherapy

Qinqin Jiang [1*], Bin Qiao [1*], Xiaohong Lin[1], Jin Cao[1], Nan Zhang[2], Huanling Guo[2], Weiwei Liu[1], Lingyu Zhu[3], Xue Xie[1], Li Wan[1], Rui Tang[1], Bing Liang[4], Dong Wang[5],Zhigang Wang[1],Yang Zhou[6], HaiTao Ran[1], Pan Li[1]*

1 Department of Ultrasound, Chongqing Key Laboratory of Ultrasound Molecular Imaging, the Second Affiliated Hospital of Chongqing Medical University, Chongqing 400010, P. R. China

2 Department of Medical Ultrasonics, The First Affiliated Hospital of Sun Yat-sen University, Guangzhou, 510080, P. R. China

3 Department of Ultrasound China-Japan Union Hospital of Jilin University, Jilin 130033, P. R. China

4 Department of Pathology, Chongqing Medical University, Chongqing, 400016, P. R. China

5 Department of Ultrasound, the First Affiliated Hospital of Chongqing Medical University, Chongqing 400010, China

6 Department of Ultrasound, the Third People's Hospital of Chengdu City, the Affiliated Hospital of Southwest Jiaotong University

*Qinqin Jiang and Bin Qiao are co-first authors who contributed equally to this work.

Corresponding authors: Pan Li, E-mail: lipan@hospital.cqmu.edu.cn.

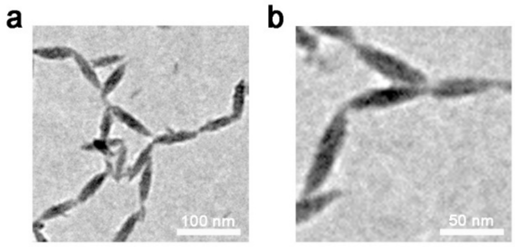

Figure S1. TEM images of FC.

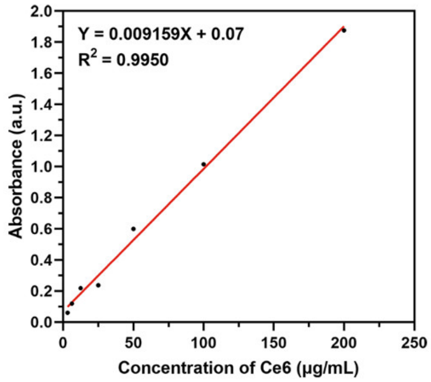

Figure S2. The relative absorbance intensity of Ce6 in the UV-vis-NIR spectrum at a wavelength of 660 nm.

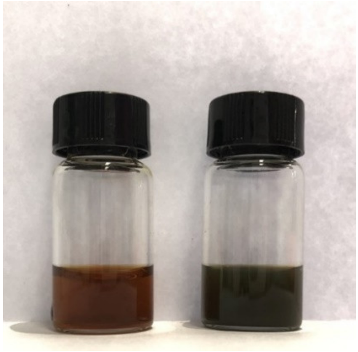

Figure S3. Digital images of Fe-PDAP (left) and MFC (right).

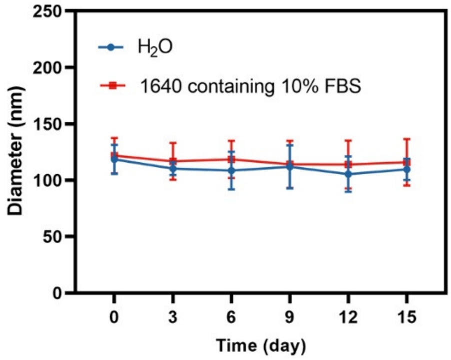

Figure S4. Size changes of MFC in H_2O and 1640 containing 10% FBS in 15 days.

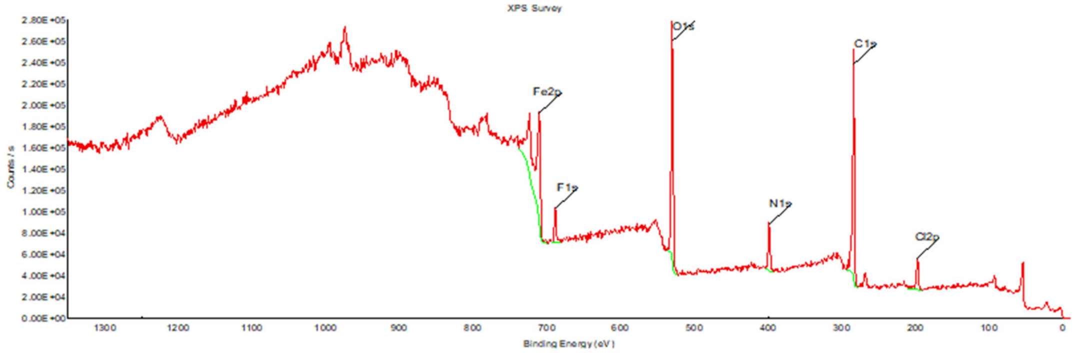

Figure S5. Full survey XPS spectrum of MFC.

Group	Hydrodynamic size (nm)	Zeta potential (mV)
MFC	126.9±2.08	-13.33±2.26
MFC+US	72.05±11.03	1.48±0.7

Figure S6. DLS results and zeta potentials of MFC with or without US irradiation (mean ±SD, n=3).

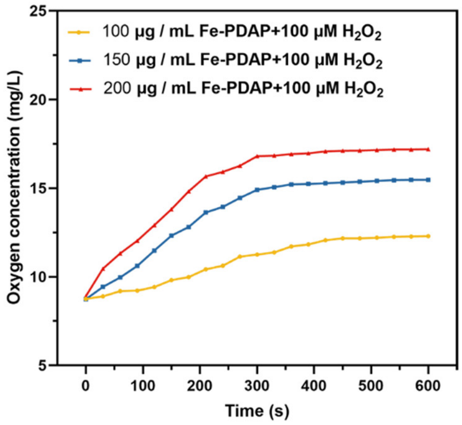

Figure S7. The production of O_2 with different concentrations of Fe-PDAP.

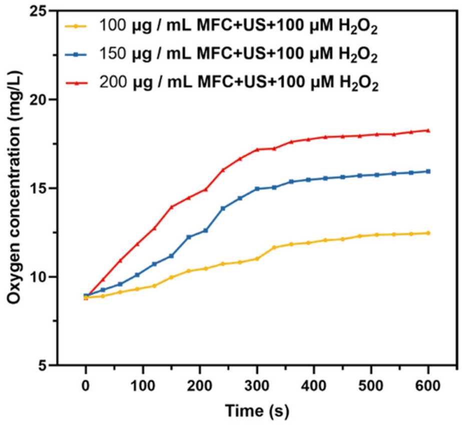

Figure S8. The production of O_2 with different concentrations of MFC after US exposure.

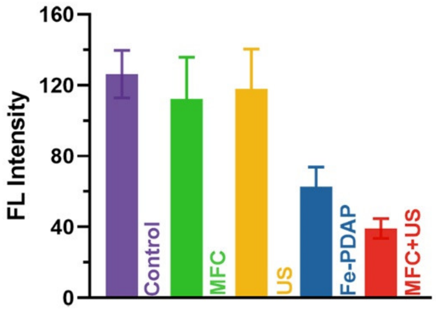

Figure S9. Fluorescence intensity of RDPP after various treatments.

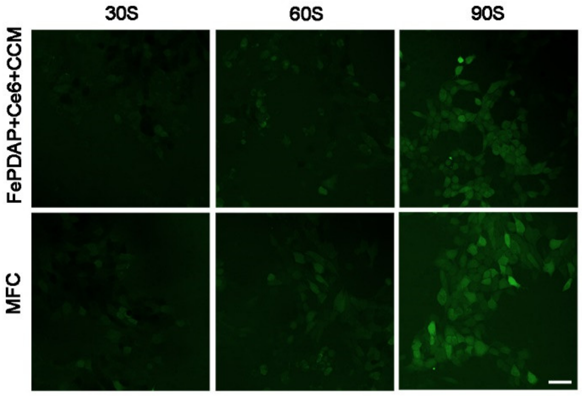

Figure S10. Intracellular ROS level observed by CLSM after different US exposure times. Scale bar: 50 μm.

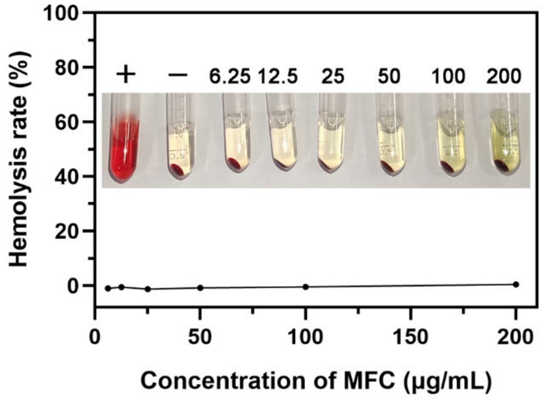

Figure S11. Hemolysis rate of RBCs treated with MFC at various concentrations. The RBC dispersed in PBS was set as a negative control, while dispersed in deionized water was set as a positive control (left two tubes). Inset: hemolysis photographs after centrifugation.

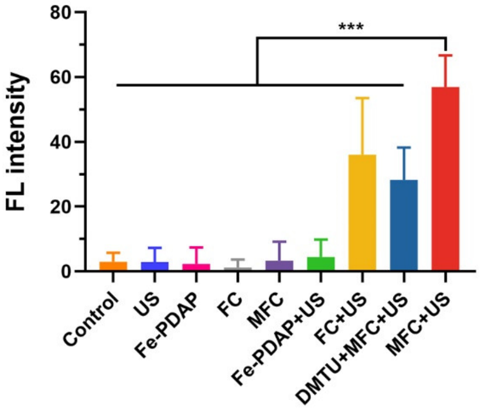

Figure S12. Quantitative analysis of DCF fluorescence intensities in 4T1 cells after various treatments observed by CLSM.

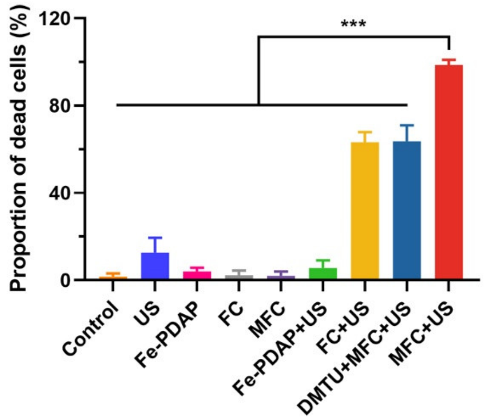

Figure S13. Quantitative analysis of 4T1 cells costained with calcein-AM (living cells, green) and propidium iodide (dead cells, red) after various treatments observed by CLSM.

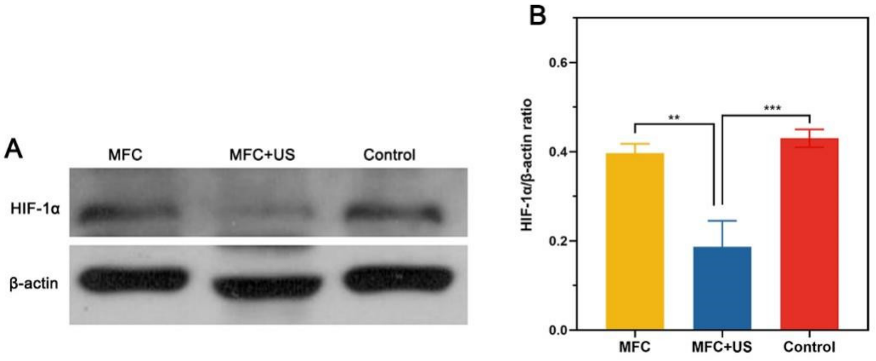

Figure S14. (A) HIF-1α expression levels in tumors after different treatments and (B) the corresponding HIF-1α/β-actin ratios.

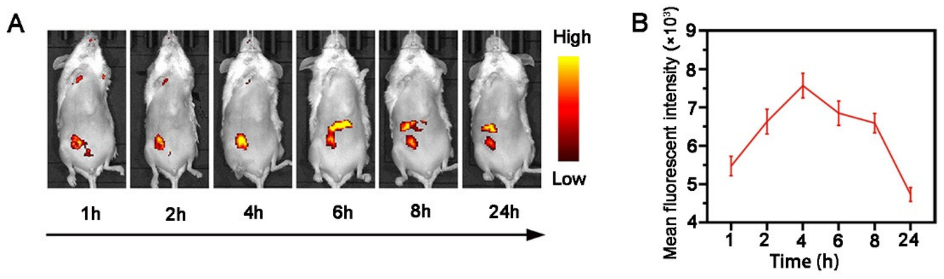

Figure S15. (A) *In vivo* fluorescence images of 4T1 tumor-bearing mice reveal the biodistribution of MFC after intravenous injection into tumor-bearing mice at different times. (B) Corresponding quantitative fluorescence signal intensity within the tumor region at different times.

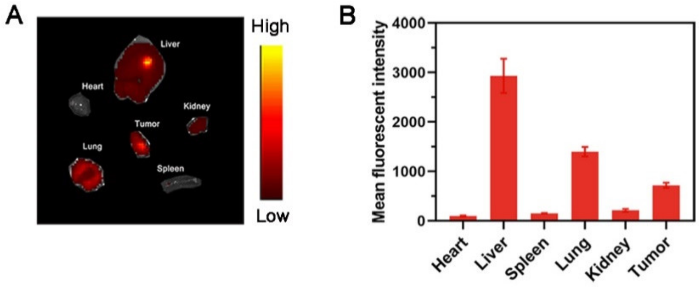

Figure S16. (A) Ex vivo fluorescence images of tumors and major organs (including the heart, liver, spleen, lungs, and kidney) 24 h after intravenous injection of MFC. (B) Corresponding quantitative biodistribution analysis of MFC in tumors and the major organs of mice 24 h post-injection.

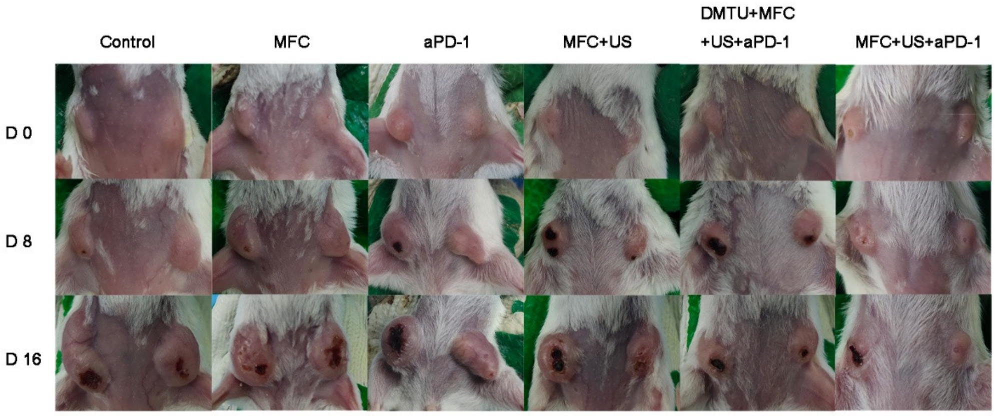

Figure S17. Representative digital photos of 4T1 tumors on both sides of BALB/c mice in different groups on days 0, 8, and 16 after different treatments.

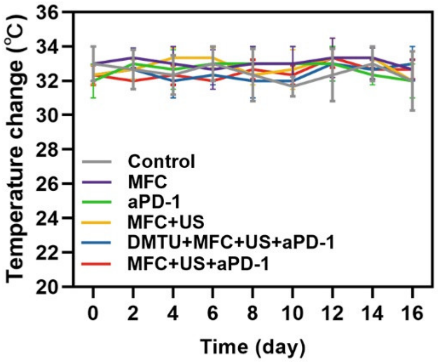

Figure S18. Time-dependent body temperature of mice.

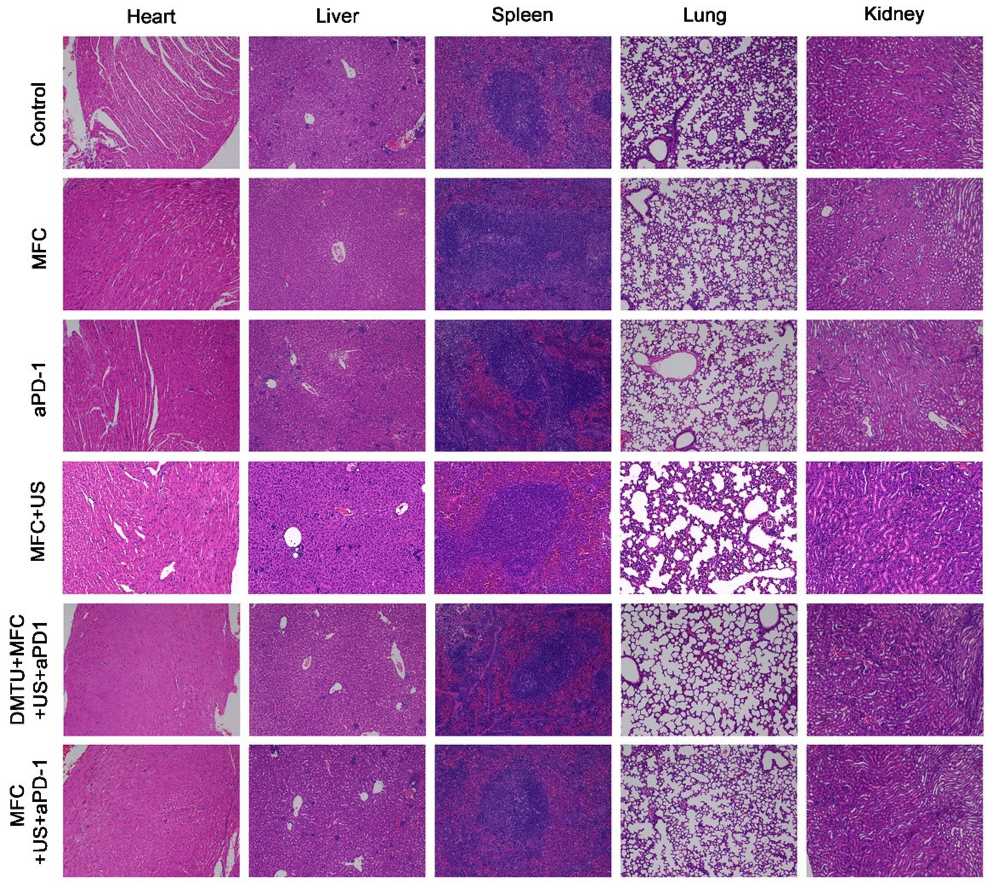

Figure S19. H&E staining of the heart, liver, spleen, lung and kidney from mice after different treatments. The magnification is 10×.

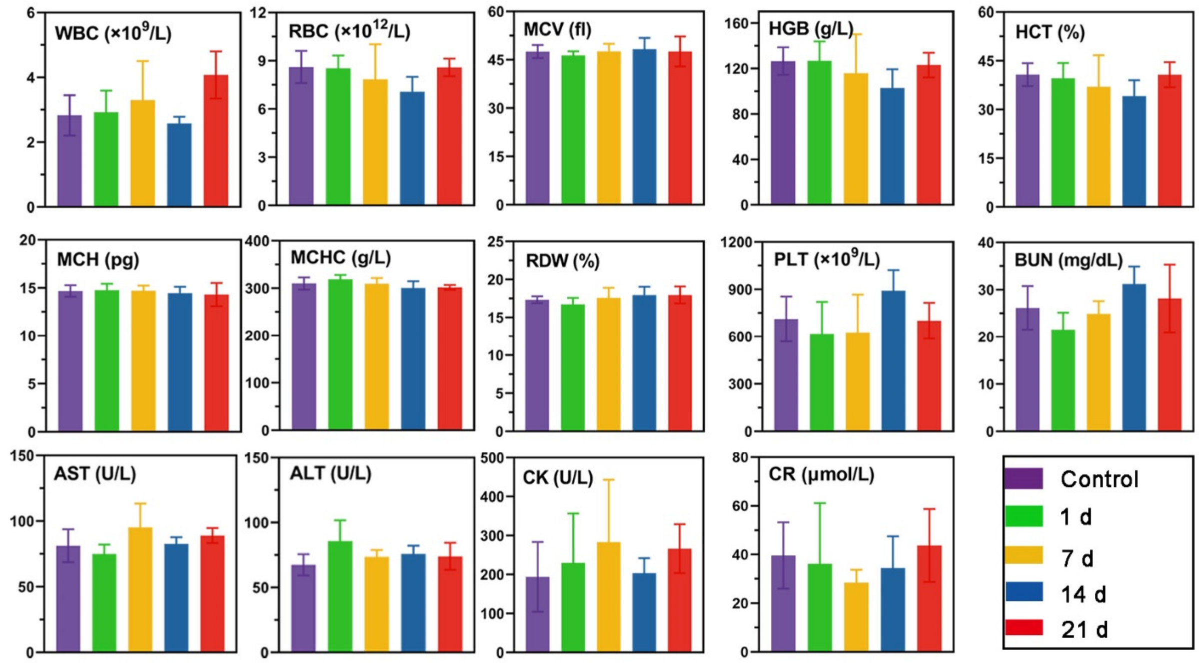

Figure S20. Routine blood tests and blood biochemistry results of mice treated with MFC at predetermined time intervals.

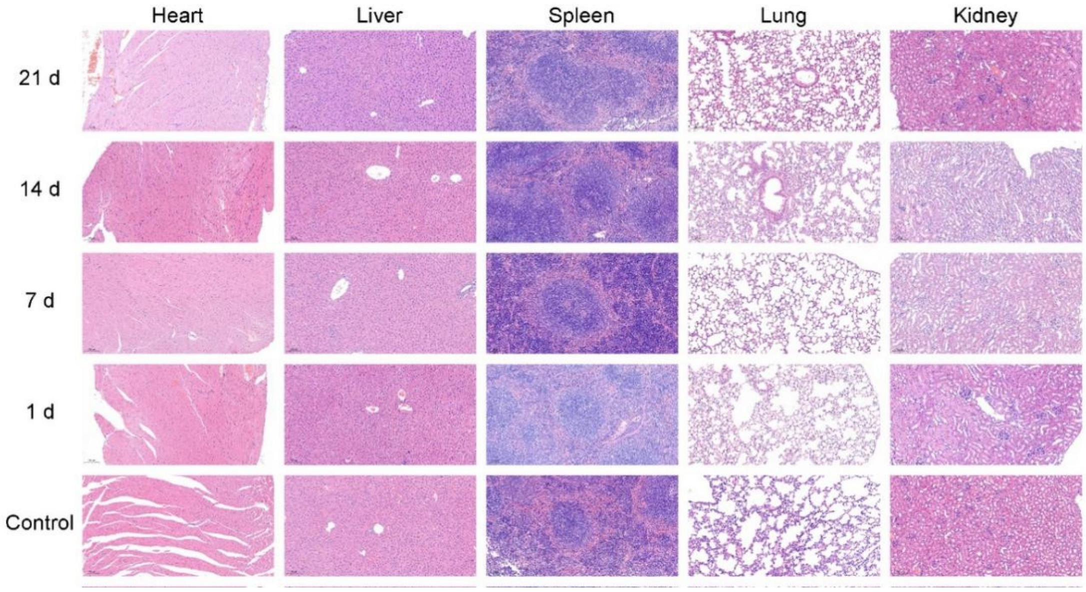

Figure S21. H&E staining of the major organs from the control group and the experimental groups 1, 7, 14, and 21 days after intravenous injection of MFC.

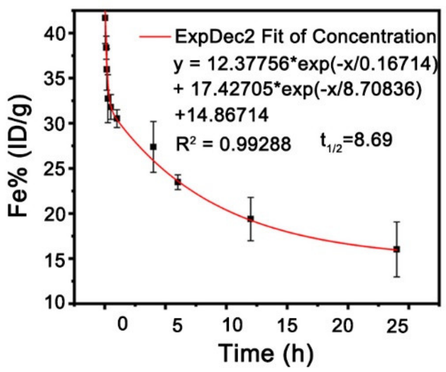

Figure S22. Blood circulation time of administered MFC determined by ICP-MS.
